# The therapeutic effect of scalp acupuncture on natal autism and regressive autism

**DOI:** 10.1186/s13020-018-0189-6

**Published:** 2018-06-15

**Authors:** Chuen Heung Yau, Cheuk Long Ip, Yuk Yin Chau

**Affiliations:** School of Chinese Medicine, Hong Kong Baptist University, Kowloon Tong, Hong Kong

**Keywords:** Natal autism, Regressive autism, Scalp acupuncture

## Abstract

**Background:**

Autism spectrum disorders (ASD) is a common disease and the incidence has been rising constantly. Acupuncture is one of the most widely used complementary and alternative medicine therapies. Despite studies had been done on the effectiveness of acupuncture on ASD children, how factors such as chronological age and the onset pattern influence the effectiveness of the therapy remains unclear. The aim of this retrospective study is to know how symptomatology of ASD alters upon the introduction of scalp acupuncture and how do age and onset type affect the effectiveness of the therapy.

**Methods:**

ASD children aged 2–11 years old were invited to join the study. In the course of the investigation, they received a total of 30 sessions of scalp acupuncture therapy. They were then evaluated to compare the performance on various aspects before and after the treatment. The influence on the therapeutic effect by factors including chorological age and onset pattern were further taken into consideration and analyzed. In addition, investigation on the relationship between allergies and onset pattern of ASD was performed by statistically analyzing the received epidemiologic data from the participants.

**Results:**

68 children with ASD participated in the study. It is found that the significant effective rate of scalp acupuncture on ASD is 97%. Scalp acupuncture can improve verbal communication problems the most while noise sensitivity improves the least. The therapeutic effectiveness decreases with increasing age and children with natal autism benefit more from acupuncture than those with regressive autism. In the latter part of the study, we observe a positive correlation between the family history of allergy and onset pattern.

**Conclusion:**

Scalp acupuncture is an effective treatment for alleviating the symptomatology of ASD. The therapeutic effectiveness is expected to be higher for those patients with natal or early onset of the disorder, and at a younger age when they receive the therapy. The study result helps to formulate an ideal regimen for ASD patients and allow therapists and parents to make appropriate expectation towards the therapeutic outcome of acupuncture. Early intervention of scalp acupuncture therapy recommended. The relationship between the family history of allergic disorder and the onset type of ASD hints that the etiologies of natal and regressive ASD are discrete. It shows a great significance in differentiating the onset pattern in carrying out clinical assessments or researches on ASD patients.

*Trial registration* This retrospective study was approved by the Committee on the Use of Human and Animal Subjects in Teaching and Research, Hong Kong Baptist University on 4th Aug 2017. The retrospectively registered number is HASC/Student/17-18/0115

**Electronic supplementary material:**

The online version of this article (10.1186/s13020-018-0189-6) contains supplementary material, which is available to authorized users.

## Background

According to World Health Organization, it was estimated that 1 in 160 children is suffering from autism spectrum disorders (ASD) worldwide and the prevalence has been rising over the past 50 years [1]. The diagnosis of ASD emphasizes few essential features, includes reciprocal social interaction, communication, and restricted and repetitive behaviors [2, 3]. In terms of onset pattern, two types of autism can be observed clinically. Children showing abnormal social development and speech delay around 1 year-old are identified as early-onset or natal autism [4]; while some children might develop normally in the first few years but lose the previously acquired skills upon the onset of autism, are known as regressive or acquired autism [5].

Since there is no definitive cure for ASD, numerous treatment methods claim to be beneficial to autistic children. Complementary and alternative medicine (CAM) treatments have been commonly used in treating ASD. Study reported that 74% of the children diagnosed with autism use one or more than one type of CAM treatments as they perceive CAM interventions to be safe and natural [6]. Among all the CAM available, scalp acupuncture has been widely used for treating ASD. In common practice of acupuncture, needles are inserted into specific points (acupoints) on the body of the patients. For scalp acupuncture, acupoints along different scalp lines or zones are selected.

Controlled trials on the effect of scalp acupuncture and electro-acupuncture on ASD patients have showed significant improvement in language comprehension and self-care ability [7, 8]. Despite the advantages of acupuncture therapy on ASD children was demonstrated in previous studies, no investigations has currently been made on how age and onset type of ASD influence the therapeutic effect of acupuncture treatment.

### Study design

A pragmatic study was conducted in a typical community based outpatient setting, from May 2010 to June 2013 at Hong Kong Baptist University Mr. & Mrs. Chan Hon Yin Chinese Medicine Specialty Clinic and Good Clinical Practice Centre. Institutional review and approval was secured by the Committee on the Use of Human and Animal Subjects in Teaching and Research, Hong Kong Baptist University (Approval number: HASC/Student/17-18/0115). The Minimum Standards of Reporting Checklist contains details of the experimental design, and statistics, and resources used in this study (Additional file 1).

## Subjects and methods

### Participants

ASD patients who consulted for acupuncture treatment at Hong Kong Baptist University Mr. & Mrs. Chan Hon Yin Chinese Medicine Specialty Clinic and Good Clinical Practice Centre were invited to join the study. Eligibility criteria included children of both gender, aged 2–11 years old, with a current medical diagnosis of ASD by a recognized specialist such as pediatrician, psychiatrist or psychologist. No cut-off exclusion criteria concerning the severity of the ASD symptoms were set.

### Therapist and treatment

Both assessment and acupuncture treatments were carried out by principal investigator (Yau Chuen Heung), who is an experienced traditional Chinese medicine practitioner specialized in acupuncture for ASD children for 18 years.

A standardized scalp acupuncture therapy was applied to all participants. Fourteen acupoints were selected based on traditional Chinese medicine theory or functional areas of the brain; including BaiHui (GV20), SiShenChong (EX-NH3), mid line of forehead, lateral line 2 of forehead, posterior lateral line of vertex, primary auditory cortex, and auditory speech area.

Participants were held and positioned properly by their parents. Their scalps were disinfected with 75% alcohol cotton ball, followed by subcutaneous insertions of 0.20 × 25 mm needles obliquely onto the acupoints, into the depth of 10 mm between aponeurosis layer and loose areolar connective tissue layer. Needles were twirled every 15 min for three times with “neutral supplementation and draining method” before all the needles were removed after an hour. Scalp acupuncture treatments were performed twice a week and the whole course consisted of 30 sessions of treatment.

### Measurement of outcome

Participants were assessed by a clinician-rated inventory on ASD-related symptoms. In light of Blatt-Kupperman index, we designed a set of rating scale for quantifying symptoms of ASD [9]. By means of a semi-structured interview with the participants and their parents, therapist would be able to score a total mark of 20, based on 5 subscale domain items rated on a 5 point scale (Table 1) that reflects the frequency and intensity of the ASD-related symptoms in aspects of social problem, verbal communication problem, behavioral problems, food selectivity and noise sensitivity.

**Table 1:** Marking criteria for score

Score	Marking criteria
0	No symptoms
1	Minimal symptoms, seldom shown
2	Mild symptoms, often shown
3	Moderate symptoms, usually shown
4	Severe symptoms, always shown

The measurement of the score was administered at the 1st and 30th session of acupuncture treatment.

Participants’ past medical history and demographic information was also recorded. Materials concerning the onset of ASD, familial and personal history of allergic diseases were also collected and manipulated. Participants who lose the previously acquired language skills were categorized into regression group, otherwise will be included into the natal group.

Therapeutic effect of acupuncture on ASD patient is evaluated by the effective rate by the following equation:

$$ {\text{Effective}}\;{\text{rate}} = \frac{{\left( {{\text{Total}}\;{\text{score}}\; 1 {\text{st}}\;{\text{acupuncture}} - {\text{Total}}\;{\text{score}}\; 3 0 {\text{th}}\;{\text{acupuncture}}} \right)}}{{{\text{Total}}\;{\text{score}}\; 1 {\text{st}}\;{\text{acupuncture}}}} \times 100\% $$

The overall therapeutic effectiveness is then concluded by categorizing patients into groups of highly effective, effective and ineffective by comparing their effective rate (Table 2).

**Table 2:** Overall therapeutic effect evaluated by effective rate

Effective rate	Significance
> 20%	Highly effective
5–20%	Effective
< 5%	Ineffective

An average improvement of one level across all domains would show the score decrease by 20% or above, which we consider to be a remarkable improvement and states the high effectiveness of the treatment. An improvement of 5% of less represents it has no improvement or only been beneficial in sole domain by a level; therefore considered to be an ineffective treatment.

### Statistical analysis

Data analyses were conducted on all treatment responders. An alpha level of 0.05 was used for all statistical tests. The mean score and standard deviation at the 1st and 30th treatment section were analyzed using paired *t* test. The effect of acupuncture on natal and regressive ASD patients of different ages was evaluated by means of independent t-test and analyses of variance (ANOVAs). Pearson Chi square tests were used to show the correlation among the type of onset of ASD, personal history of allergic disorder and family history of allergy. All the calculations were performed on software IBM SPSS Statistics (Windows, version 21).

## Results

### Participant characteristics

Among 68 patients with autism spectrum disorders, there were 11 female and 57 male. Ages ranged from 2 to 10 years old. The oldest child was 10 years and 7 months old while the youngest one was 2 years and 1 month old. 47 (69%) natal autism cases and 21 (31%) regressive autism cases were included.

### Comparison of clinical manifestation before the 1st and after the 30th acupuncture treatment on both natal and regressive autism

**Fig. 1:**
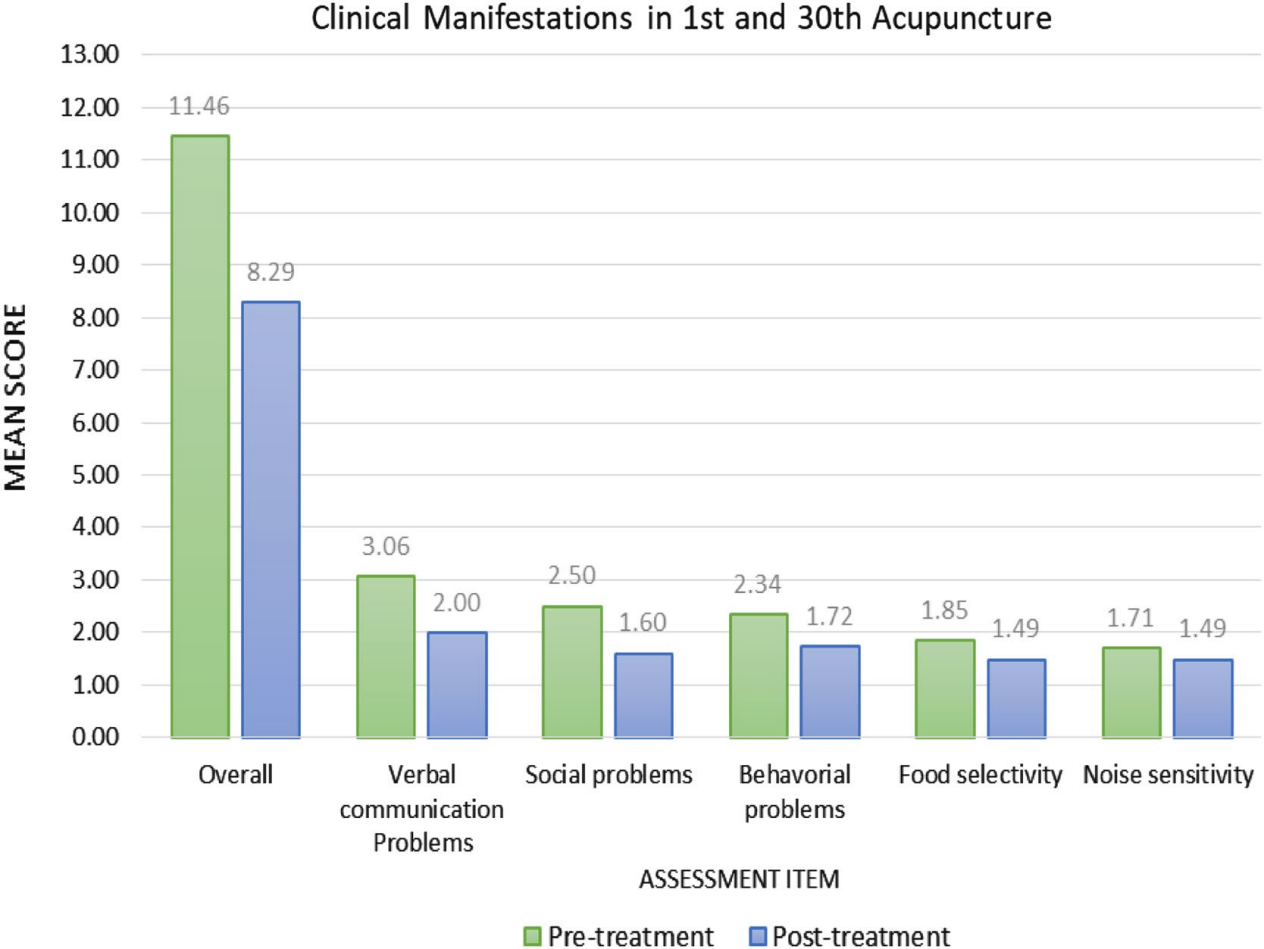
Clinical manifestations in 1st and 30th acupuncture

In the first section, we tried to investigate the effect of acupuncture on ASD patients, and the results are shown in Table 3 and Fig. 1. Before acupuncture, item of verbal communication problems scored the highest mark with the mean score of 3.06, followed by social problems and behavioral problems scoring 2.50 and 2.34, respectively. While food selectivity and noise sensitivity scored the least marks of 1.85 and 1.71, respectively.

**Table 3:** Comparison of clinical manifestation before the 1st and after the 30th acupuncture treatment in natal and regressive autism of different age

Items	Pre-treatment		Post-treatment		Percentage change (-%)	P-value
	Mean	SD	Mean	SD		
All	11.46	2.37	8.29	2.03	27.60	0.000
Verbal communication problems	3.06	1.01	2.00	0.90	34.62	0.000
Social problems	2.50	0.78	1.60	0.69	35.88	0.000
Social problems	2.34	1.07	1.72	0.77	26.42	0.000
Social problems	1.85	0.70	1.49	0.63	19.84	0.000
Noise sensitivity	1.71	0.88	1.49	0.76	12.93	0.003

The scores of five symptoms decreased after the thirtieth acupuncture, and the improvement is significant (p < 0.05). Among all domains, improvement made on social problems (− 35.9%) and verbal communication problems (− 34.6%) have been most prominent. Other items such as behavioral problems (− 26.4%), food selectivity (− 19.8%) and noise sensitivity (− 12.9%) showed relatively less effective towards acupuncture treatment.

### Comparison of the change in clinical manifestation after acupuncture between natal and regressive autism of different age

#### Verbal communication problems

Table 4 shows the data about the communication performance in natal and regressive ASD children of different age. It was found that p-value comparing the score of pre-treatment and post-treatment in all ages of natal group was smaller than 0.05; whereas for regressive autism, age groups other than five or above showed a p-value greater than 0.05. Results indicates a general significant improvement in verbal communication problems for both onset types across all age groups after acupuncture therapy.

**Table 4:** Comparison of verbal communication problems before the 1st and after the 30th acupuncture treatment in natal and regressive autism of different age

Age (year)	Natal						Regressive					
	Pre-treatment			Post-treatment			Pre-treatment			Post-treatment		
	n	Mean	SD	Mean	SD	p-value	n	Mean	SD	Mean	SD	p-value
All	47	2.87	0.99	1.87	0.80	0.000	21	3.48	0.93	2.29	1.06	0.000
2	8	3.25	1.16	2.25	0.89	0.001	4	4.00	0.00	2.75	0.50	0.015
3	14	2.93	0.83	1.57	0.76	0.000	8	3.63	0.74	2.25	1.04	0.008
4	17	2.82	1.01	1.88	0.78	0.000	5	3.00	1.00	2.20	0.84	0.016
5	8	2.50	1.07	2.00	0.76	0.033	4	3.25	1.50	2.00	1.83	0.141

More specifically, natal onset ASD children with age of 2 to 3 improved the most and their mean scores were reduced by 1.2–1.0, while the score of 4 years old children decreased by 0.94. The children above 5 years old improved the least and their score decrease 0.5 only. In regressive group, both scores of children of 2 years old and 3 years old reduced by 1.25 and 1.38, respectively while that of children of 4 years old decreased by 0.8. The score of children with age above five reduced a mark of 1.25. Concerning the problems in verbal communication in both natal and regression ASD, we observed a better improvement in children of younger age to receive acupuncture treatment.

#### Social problems

Table 5 shows the improvement in social problems in natal and regressive ASD children of different age. In natal onset group, p-value of all age groups are less than 0.05. In group of regressive autism, only the p-value of age above five is greater than 0.05, while the remaining groups obtained the value smaller than 0.05. Patients performed significantly better in social interaction after acupuncture treatment.

**Table 5:** Comparison of social problems before the 1st and after the 30th acupuncture treatment in natal and regressive autism of different age

Age (year)	Natal						Regressive					
	Pre-treatment			Post-treatment			Pre-treatment			Post-treatment		
	n	Mean	SD	Mean	SD	p-value	n	Mean	SD	Mean	SD	p-value
All	47	2.47	0.83	1.53	0.72	0.000	21	2.57	0.68	1.76	0.62	0.000
2	8	2.63	0.52	1.25	0.46	0.001	4	3.00	0.00	2.00	0.00	0.015
3	14	2.36	1.01	1.21	0.43	0.000	8	2.75	0.46	1.88	0.64	0.000
4	17	2.59	0.80	1.82	0.88	0.003	5	1.80	0.45	1.00	0.00	0.016
5	8	2.25	0.89	1.75	0.71	0.033	4	2.75	0.96	2.25	0.50	0.182

The score of natal ASD children with age of 2 and 3 decreased by 1.38 and 1.15, respectively while the score of 4 year-old children fell by 0.77. With the least decrease, the score of “above 5 year-old” group reduced by 0.50. The pattern of improvement across different age groups in regressive autism group is similar to that of natal group. The improvement in score of children with age of 2 and 3 are 1.00 and 0.87, respectively. The score of children with age of 4 fell by 0.80. The score of children with age of 5 or above reduced the least of 0.50 marks.

#### Behavioral problems

Table 6 shows how behavior problems changed in natal and regressive ASD patients of different age. In group of natal autism, p-value for the 5 year-old group is larger than 0.05 while the rest of the age groups are lesser than 0.05. On the contrary, no groups obtain a p-value below 0.05 in regressive autism. However, in the calculation, the p-value in paired t-test in regressive ASD regardless of the age group is below 0.05. Therefore, we suggest that improvement in behavioral problems is significant in both natal and regressive autistic children in general, despite it is reasonable to expect that natal ASD individual are more likely to make more remarkable progress in behavior aspect when compared with regressive individuals.

**Table 6:** Comparison of behavioral problems before the 1st and after the 30th acupuncture treatment in natal and regressive autism of different age

Age (year)	Natal						Regressive					
	Pre-treatment			Post-treatment			Pre-treatment			Post-treatment		
	n	Mean	SD	Mean	SD	p-value	n	Mean	SD	Mean	SD	p-value
All	47	2.38	1.09	1.72	0.77	0.000	21	2.24	1.04	1.71	0.78	0.004
2	8	2.88	1.13	1.88	0.64	0.007	4	2.00	0.82	1.25	0.50	0.058
3	14	1.86	0.95	1.50	0.65	0.014	8	2.50	1.20	2.13	0.99	0.197
4	17	2.59	1.00	1.82	0.88	0.001	5	1.80	1.10	1.60	0.55	0.621
5	8	2.38	1.30	1.75	0.89	0.180	4	2.50	1.00	1.50	0.58	0.092

As shown in Table 6, in natal autism group, the score of children with age of 2 fell by 1.00 while that of children with age of 3 reduced by 0.36. The scores of children who are 4 years old and above 5 years old decreased by 0.77 and 0.63, respectively.

#### Food selectivity

In Table 7, it shows the data about food selection problems in the patients. p-values of 2 groups in natal autism were smaller than 0.05 and they were the groups of “2 year-old to 2 year-old and 11 months” and group of “3 year-old to 3 year-old and 11 months”. The p-value of group “4 year-old to 4 year-old and 11 months” and group of “above 5 year-old” was greater than 0.05. Besides, all p-value of groups in regressive autism cannot show a value smaller than 0.05. Despite the difference within each age groups might not be statistically significant, when we consider the overall p-value across all age groups of natal and regressive group, p-value is below 0.05, indicating an overall significance effect of acupuncture in different onset types.

**Table 7:** Comparison of food selectivity before the 1st and after the 30th acupuncture treatment in natal and regressive autism of different age

Age (year)	Natal						Regressive					
	Pre-treatment			Post-treatment			Pre-treatment			Post-treatment		
	n	Mean	SD	Mean	SD	p-value	n	Mean	SD	Mean	SD	p-value
All	47	1.85	0.69	1.45	0.62	0.000	21	1.86	0.73	1.57	0.68	0.030
2	8	1.75	0.71	1.00	0.00	0.020	4	1.50	0.58	1.00	0.00	0.182
3	14	1.64	0.63	1.29	0.47	0.009	8	1.88	0.83	1.88	0.83	0.080
4	17	2.06	0.75	1.71	0.69	0.055	5	2.40	0.55	1.80	0.45	0.208
5	8	1.88	0.64	1.63	0.74	0.170	4	1.50	0.58	1.25	0.50	0.391

It shows that the improvement of food selectivity fell with increasing age in natal group. The improvement has been most remarkable in age group of 2 years old with a score decrement of 0.75. The drop in score of children with age of 3 and 4 are both 0.35; followed by that of children with age of above 5 scoring a 0.25-point decrease.

#### Noise sensitivity

In Table 8, it shows the score indicating noise sensitivity issues. In the item of noise sensitivity, only p-values of 4 year-old children with natal autism and 3 year-old children with regressive autism were smaller than 0.05 and the p-values of remaining children were larger than 0.05.

**Table 8:** Comparison of food selectivity before the 1st and after the 30th acupuncture treatment in natal and regressive autism of different age

Age (year)	Natal						Regressive					
	Pre-treatment			Post-treatment			Pre-treatment			Post-treatment		
	n	Mean	SD	Mean	SD	p-value	n	Mean	SD	Mean	SD	p-value
All	47	1.74	0.90	1.53	0.78	0.024	21	1.62	0.86	1.38	0.74	0.056
2	8	2.13	0.89	1.75	1.04	0.351	4	1.75	0.96	1.00	0.00	0.215
3	14	1.43	0.65	1.21	0.58	0.083	8	1.63	0.92	1.63	0.92	0.011
4	17	2.06	1.14	1.65	0.86	0.029	5	1.60	0.89	1.20	0.45	0.178
5	8	1.63	0.52	1.63	0.52	NA	4	1.50	1.00	1.50	1.00	0.215

For natal group, the score of 4 years old children dropped the most with a descent of 0.41, followed by age of 2 which decreased 0.38. Children with age of 3 score 0.22 less. Sound sensitivity in children above 5 years old shows no changes upon the treatment. In regressive autism group, the score of 2 years old children decreased by 0.75 while 4 years old decreased by 0.4. The score of children with age of 3 and above 5 remains constant after all the treatment sessions.

#### Overall score and effect

**Fig. 2:**
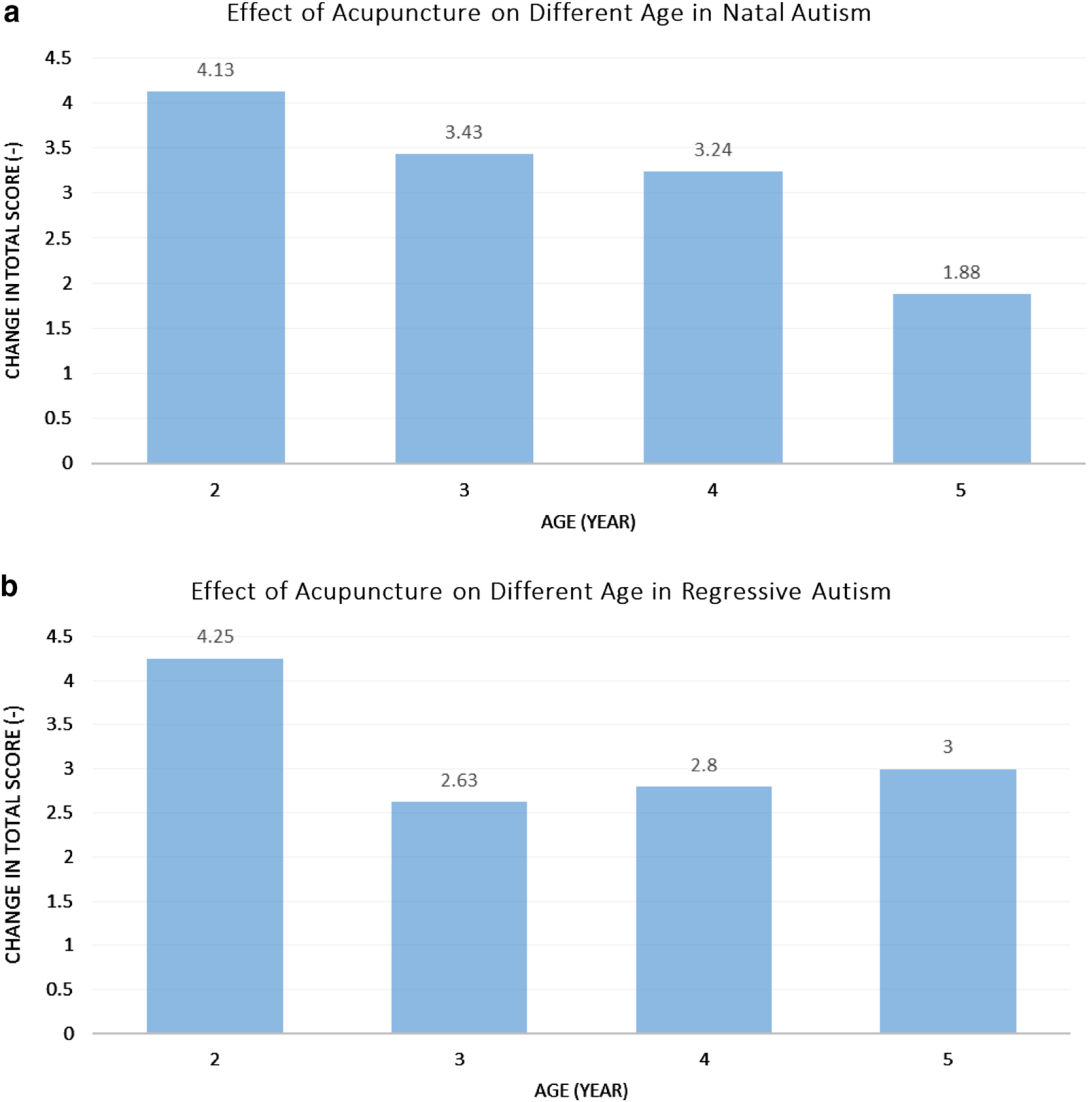
**a** The change in total score on different age groups after 30th acupuncture in natal autism. **b** The change in total score on different age groups after 30th acupuncture in regressive autism

From Table 9, the overall therapeutic effect of acupuncture on natal and regressive autism along with the age of the patients is observed. P-values of all the groups in both natal autism and regressive autism were smaller than 0.05. Therapeutic effect of acupuncture is significant on both natal and regressive ASD patients of all the age groups. From Fig. 2a, the overall effectiveness of scalp acupuncture on natal ASD patients dropped with the increment of the age. In Fig. 2b shows the overall effectiveness on regressive group is highest at the age of 2, tumbled to the lowest at 3 years old and then retained a slight and steady increase when the patients are older.

**Table 9:** Comparison of Overall manifestation before the 1st and after the 30th acupuncture treatment in natal and regressive autism of different age

Age (year)	Natal						Regressive					
	Pre-treatment			Post-treatment			Pre-treatment			Post-treatment		
	n	Mean	SD	Mean	SD	p-value	n	Mean	SD	Mean	SD	p-value
All	47	11.32	2.49	8.11	2.09	0.000	21	11.76	2.10	8.71	1.87	0.000
2	8	12.25	1.91	8.13	1.25	0.000	4	12.25	1.26	8.00	0.00	0.007
3	14	10.21	2.36	6.79	1.25	0.000	8	12.38	2.20	9.75	2.49	0.002
4	17	12.12	2.62	8.88	2.29	0.000	5	10.60	2.70	7.80	1.30	0.025
5	8	10.63	2.33	8.75	2.60	0.001	4	11.50	1.73	8.50	1.29	0.001

From Table 10, among 68 children with ASD, 51 of them had highly significant improvement while 15 of them had significant improvement. Only 2 of them did not have significant improvement. The highly significant rate was 75% and the significant rate was 22%. The total significant rate was 97%.

**Table 10:** Overall effect of scalp acupuncture on ASD

	n	Percentage (%)
Total	68	100
Highly significant	51	75
Significant	15	22
Not significant	2	3

#### Relationship between allergy and ASD

17 (25%) participants had shown various degree of allergic disorders such as allergic rhinitis, asthma and eczema, while the remaining (75%) showed not history or relevant disorders. Concerning the family history of allergy-related disease, 29.4% (n = 20) participants’ father or mother had a history of respiratory or dermatologic allergic disorder, while the rest of 70.6% (n = 48) participants’ parents did not.

**Fig. 3:**
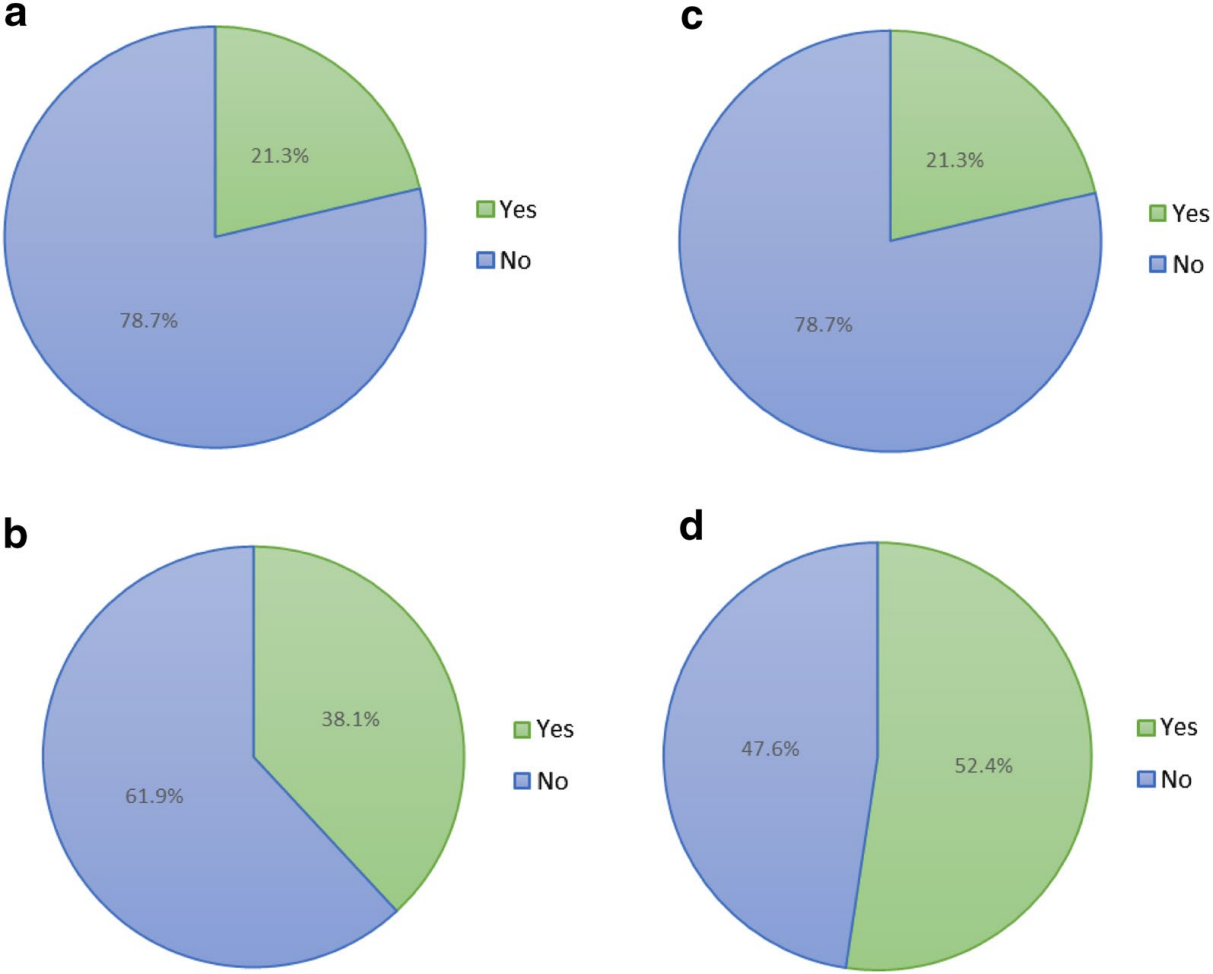
**a** History of allergic disorder in natal ASD. **b** History of allergic disorder in regressive ASD. **c** Family history of allergic disorder in natal ASD. **d** Family history of allergic disorder in regressive ASD

Figure 3a, b represents the percentage of patients having allergic disorder and Graph Fig. 3c, d shows the percentage of patients having a family history of allergic disorder. By means of Pearson’s Chi square tests, the interrelations among family history, personal allergies, and onset type (natal or regressive) were evaluated. Results show a significant correlation between family and personal history of allergy diseases (p = 0.000), and also between family history of allergies and the onset type of ASD (p = 0.000). However, no significant correlation between personal history of allergies and the type of ASD can be statistically drawn (p = 0.293).

## Discussion

In the first section, we looked into the baseline symptomatology of the participants before acupuncture treatment. Considering the background score across the measuring domains, we observed that verbal communication problems and social problems showed the greatest improvement while food selectivity and noise sensitivity showed the least progress.

Age is a predictor for treatment outcome. Studies have shown that patients of 5 years old or younger benefit from a more promising treatment outcome from applied behavior analysis treatment [10–12]. To date, there was no research on how the age of the children affect the therapeutic outcome of acupuncture to ASD. Yet it is well perceived clinically that younger patients are more responsive to scalp acupuncture. Our investigation reassures that sooner the treatment starts; better the therapeutic effect can be achieved. Thus, early intervention of acupuncture is encouraged for ASD patients, especially those with commanding speech and social problems. It is important to state that although therapeutic effect reduces with age, older patients still benefit from acupuncture.

Scalp acupuncture is effective with statistical significance on treating both natal and regressive autism. Our clinical experience tells natal ASD patient generally benefit more from acupuncture than regressions ASD. We manipulated a series of independent t-test to investigate how natal and regressive ASD patients manifest and response to treatment differently. Despite natal ASD patients show a better response to acupuncture on average, the calculation displays no significant discrepancies (p > 0.05) between the two onset types in pre-treatment score or change in score across all items except verbal communication problems. Analysis reveals that that natal group performs significantly better in language use than regressive group (p = 0.021). We therefore reasonably propose that despite successful acquisition of language skills prior to the regression does not provide any protective values; it assists a more rapid re-mastering of precedent learnt abilities or brings about better progress upon effective treatments or recovery.

In the second part of the study, we looked into the relationship between allergy and ASD. Strong correlation between familial and maternal atopic history and ASD had been well-recognized [13–16]. Furthermore, Molloy [17] revealed familial autoimmune disease such as thyroid disease is a significant risk factor to the regressive onset of ASD. Our investigation hints that familial atopies apart from thyroid disease, such as asthma, rhinitis and eczema, also exhibit similar relationships. Thus individual with a family history of allergies are more likely to develop into regressive rather than natal ASD.

The reason of late onset of autism remains unclear and we still cannot completely rule out the possibilities that environment factors including vaccination such as measles, mumps and rubella (MMR) shots, might contribute to the occurrence of regressive ASD. On the other hand, we clinically observe abnormal immune response status on ASD individuals. It is demonstrated as a vigorous response to mosquito bites or frequent cold and fever experienced by patients. It is therefore postulated that attenuated immune system might be involved in the onset or the presentation of ASD.

The biological mechanism beneath the working of acupuncture on ASD patients remains unclear. We believed that acupuncture on scalp can stimulate and activate the release of neuron transmitter and therefore assist in “rewiring” the defective neuron pathways. Experiments on mouse models suggested that the stimulation at specific scalp areas can increase the expression of a postsynaptic density protein 95 (PSD-95) and activate nitric oxide synthase (NOS), result in improving learning memory ability and intelligence respectively [18, 19]. Different areas and lines can be drawn on the scalp as a projection of functional areas of cerebrum according to reflexology. For example, midline and the lateral line 2 of forehead are in response to the prefrontal cortex of frontal lobes; posterior lateral line of vertex in response to posterior parietal lobe; auditory speech area is in response to the Brodmann area 22, etc. Acupuncture at these areas was performed in order to stimulate the activity of the corresponding cerebral function.

There are few limitations in the present study. Firstly, 68 patients joined the study and a larger size of sample is more preferable. Secondly, it is desirable to obtain laboratory data such as functional magnetic resonance imaging (fMRI) for spotting any alteration in brain function throughout the course of acupuncture treatment.

## Conclusion

ASD manifestations of some aspects such as verbal communication, social, and behavioral problems obtained a highly significant improvement upon the introduction of acupuncture; whereas domains of food selectivity and auditory sensitivity benefit less in the process. Age and the onset type are predictors for the therapeutic effect of acupuncture. We shall expect better therapeutic effect on natal onset ASD children with a younger age of two. Therefore, early intervention is always encouraged for ASD children.

Due the discrepancies between the effect of acupuncture on natal and regressive onset ASD, we postulated deviation in the etiology or the mechanisms between the two onset types. Our result showing the correlation between family history of atopy and onset type coheres with our hypothesis stating the difference in nature of natal and regressive ASD. Yet much more effort is required to reveal the underlying mechanisms for autism.

Despite the rapid development of modern science, the incidence rate of ASD shows no sign of decline and remains to be an incurable disorder. Only if we could reveal factors or incidences that would cause or induce the onset of the ASD, i.e., regressive ASD, preventive measure or effective treatments could be applied. Whilst the mechanism of ASD remains unsettled, we should remain skeptical about the use of medical manipulations such as vaccination on infants and young kids.

## Additional file

**Additional file 1.** Minimum Standards of Reporting Checklist.

## Abbreviation

ASD: autism spectrum disorder
CAM: complementary and alternative medicine
fMRI: functional magnetic resonance imaging
MMR: measles—mumps—rubella
NOS: nitric oxide synthase
PSD-95: postsynaptic density protein 95

## References

[1] World Health Organization (WHO. Autism spectrum disorders: Fact sheet. Accessed 21 May 2017. 2017.

[2] World Health Organization (1992). The ICD-10 classification of mental and behavioural disorders: clinical descriptions and diagnostic guidelines.

[3] American Psychiatric Association (2013). Diagnostic and statistical manual of mental disorders (DSM-5^®^).

[4] Ozonoff S, Heung K, Byrd R, Hansen R, Hertz-Picciotto I (2008). The onset of autism: patterns of symptom emergence in the first years of life. Autism Res..

[5] Hoshino Y, Kaneko M, Yashima Y, Kumashiro H, Volkmar FR, Cohen DJ (1987). Clinical features of autistic children with setback course in their infancy. Psychiatry Clin Neurosci.

[6] Hanson E, Kalish LA, Bunce E, Curtis C, McDaniel S, Ware J, Petry J (2007). Use of complementary and alternative medicine among children diagnosed with autism spectrum disorder. J Autism Dev Disord.

[7] Allam H, Eldine NG, Helmy G (2008). Scalp acupuncture effect on language development in children with autism: a pilot study. J Altern Complement Med..

[8] Wong VC, Chen WX, Liu WL (2010). Randomized controlled trial of electro-acupuncture for autism spectrum disorder. Altern Med Rev..

[9] Alder E (1998). The Blatt-Kupperman menopausal index: a critique. Maturitas..

[10] Fenske EC, Zalenski S, Krantz PJ, McClannahan LE (1985). Age at intervention and treatment outcome for autistic children in a comprehensive intervention program. Anal Interv Dev Disabil..

[11] Rogers SJ (1996). Brief report: early intervention in autism. J Autism Dev Disord.

[12] Harris SL, Handleman JS (2000). Age and IQ at intake as predictors of placement for young children with autism: a four-to six-year follow-up. J Autism Dev Disord.

[13] Comi AM, Zimmerman AW, Frye VH, Law PA, Peeden JN (1999). Familial clustering of autoimmune disorders and evaluation of medical risk factors in autism. J Child Neurol.

[14] Sweeten TL, Bowyer SL, Posey DJ, Halberstadt GM, McDougle CJ (2003). Increased prevalence of familial autoimmunity in probands with pervasive developmental disorders. Pediatrics.

[15] Croen LA, Grether JK, Yoshida CK, Odouli R, Van de Water J (2005). Maternal autoimmune diseases, asthma and allergies, and childhood autism spectrum disorders: a case-control study. Arch Pediatr Adolesc Med.

[16] Atladóttir HÓ, Pedersen MG, Thorsen P, Mortensen PB, Deleuran B, Eaton WW, Parner ET (2009). Association of family history of autoimmune diseases and autism spectrum disorders. Pediatrics.

[17] Molloy CA, Morrow AL, Meinzen-Derr J, Dawson G, Bernier R, Dunn M, Hyman SL, McMahon WM, Goudie-Nice J, Hepburn S, Minshew N (2006). Familial autoimmune thyroid disease as a risk factor for regression in children with autism spectrum disorder: a CPEA study. J Autism Dev Disord.

[18] Zhang XJ, Wu Q (2013). Effects of electroacupuncture at different acupoints on learning and memory ability and PSD-95 protein expression on hippocampus CA1 in rats with autism. Zhongguo zhen jiu = Chin Acupunct Moxib..

[19] Cui L, Sun GJ, Zhou H, Du YJ (2009). Influence of pre-stimulation with acupuncture and moxibustion on learning and memory ability and the activity of sod, nos in hippocampal area of alzheimer disease model rats. J Hubei Univ Chin Med..

