# Supplementary material for: The therapeutic effect of scalp acupuncture on natal autism and regressive autism
Source: Chin Med. 2018 Jun 15;13:30. doi: 10.1186/s13020-018-0189-6 (PMC6003091; doi:10.1186/s13020-018-0189-6)
Supplement: Supplementary file 1 — Additional file 1. Minimum Standards of Reporting Checklist. [file 13020_2018_189_MOESM1_ESM.pdf]

BioMed Central
The Open Access Publisher

Minimum Standards of Reporting Checklist

BioMed Central advocates full and transparent reporting. Please ensure that your paper provides the information requested below where applicable. On submitting your paper you will be asked to confirm you have included this information, or give reasons for any instances where it is not made available. You will also be asked to upload this file and it should be cited in the Methods section.

Experimental design and statistics

The following information should be included in the Methods section and inserted in the table below:

Question	Answer
1. The exact sample size (n) for each experimental group/condition (as a number, not a range). Include details of a power analysis if done, or any other relevant considerations that determined the choice of sample size. For n < 6, individual data values should be shown rather than summary statistics alone.	The sample size is 68; 47 of them is natal Autism Spectrum Disorder (ASD) , 21 is regressive. Age groups are also set, The number of individuals in age group of 2 years old, 3 years old, 4 years old and above 5 years old are 12,22,22 and 12 respectively. No special consideration of power analysis on the choice of sample size.
2. A description of sample collection that enables the reader to understand whether the samples represent technical or biological replicates, and an explanation of inclusion/exclusion criteria if samples or organisms were excluded from the analysis.	The sample collection are from patients who volunteered to receive acupuncture therapy and recorded for research purpose. No cases of exclusion once they fulfill age and diagnostic criteria. Inclusion criteria was set to maintain independent variables.
3. How samples/ organisms were allocated to experimental groups and processed, and full details of the randomisation procedure used (if relevant).	Not applicable for our study as it involves no randomisation in the process.
4. For sample assessment by human investigators, a statement on whether the investigator was blinded to group assignment and outcome assessment, and how this blinding was achieved and evaluated (if relevant).	Not applicable for our study as participants received standardised treatment.

5. How many times each experiment shown was replicated and an indication of the extent of variation from experiment to experiment.	No replication of result has been made.
6. Information on the statistical methods and measures used. It should be clear whether the tests are one-sided or two-sided, whether there are adjustments for multiple comparisons, whether medians or means are being shown, whether error bars are standard deviations (SD), standard error of mean (SEM) or confidence intervals.	Paired t-tests were used to show the effect of the treatment. Independent t-test and analyses of variance (ANOVAs) were used to correlate the factors such as age and the onset type with the effect of treatment. Pearson Chi-square tests were used to show the correlation between ASD and allergies. The tests are one sided with a alpha level of 0.05. means are shown in the table or figures while error bars have not be used in the presentation.
7. A justification for the appropriateness of statistical tests used to assess significance. Do the data meet the assumptions of the tests? Is there an estimate of variation within each group of data, and is the variance similar between groups that are being statistically compared?<br>In addition, information essential to interpreting the data presented should be made available in the figure and table legends.<br>If the study involves health interventions for human participants, please refer to the relevant reporting guidelines from the EQUATOR Network, and the Biosharing Portal for reporting checklists for biological and biomedical research, where applicable.	The data meets the assumption of the tests.<br>Figure and table legends were drawn to facilitate interpretation.

Research involving humans

If your research involved humans, please confirm you have adhered to the relevant reporting guideline from the EQUATOR Network, and included the completed checklist as an additional file with your submission:

	**Answer** (page and line number inserted/Not applicable for my study)
- I have followed the relevant reporting for my study type, and included a populated checklist with my submission<br>- Not applicable for my study	We have followed the relevant relevant reporting for my study type, and included checklist with my submission.on the third line of page 3.

BioMed Central
The Open Access Publisher

Resources

A description of all resources used should be included in the Methods section, with enough information to allow them to be uniquely identified. The table below should be completed with confirmation that this was done (i.e. included in the Methods section) or is not applicable. If this has not been completed, but is applicable, you should contact the journal editorial staff before proceeding.

	**Answer** (page and line number inserted/Not applicable for my study)
•Antibodies: report source, catalogue code, characteristics, dilutions and how they were validated for the system under study.	Not applicable for our study.
•Cell lines: report source, whether identity has been authenticated and whether tested for mycoplasma contamination. We encourage researchers to check the NCBI database for contamination of cell lines.	Not applicable for our study.
•Organisms: report source, species, strain, sex, age, husbandry, inbred and strain characteristics of transgenic and mutant animals.	Not applicable for our study.
•Tools (software, databases and services): report standard tool name, provider and version number, if available. For antibodies, model organisms (mice, zebrafish and flies) and tools, authors are strongly encouraged to cite Research Resource Identifiers (RRIDs). To do so, please go to the Resource Identification Portal to search for your research resource and insert the reference text into your Methods section.	IBM SPSS Statistics (Windows, version 21)

Availability of data and materials

The table below should be completed with confirmation that this was done (i.e. included in the Methods section) or is not applicable.

	**Answer** (page and line number inserted/Not applicable for my study)
All datasets on which the conclusions of the paper rely must be either deposited in publicly available repositories (where available and ethically appropriate) or presented in the main paper or additional supporting files, in machine-readable format whenever possible. If authors are unable to fulfil this requirement, they should contact journal editorial staff, after checking our list of Recommended Repositories.	School of Chinese Medicine's membership page.
Links to deposited datasets, or datasets in additional files, should be explicitly referenced in a section entitled "Availability of Data and Materials". Guidance on where to deposit your data can be found on the Availability of Data and Materials policy page.	Not applicable for our study
If computer code was used to generate results that are central to the paper's conclusions, include a statement in the "Availability of data and materials" section to indicate how the code can be accessed. Include version information and any restrictions on availability. For deposited data and published code, a full reference with an accession number, doi or other unique identifier should be included in the reference list.	Not applicable for our study
If reproducible materials are generated as a result of the research (for example new animal mutants), a statement on their availability should be included.	Not applicable for our study
